# The Placental Role in Gestational Diabetes Mellitus: A Molecular Perspective

**DOI:** 10.17925/EE.2024.20.1.5

**Published:** 2024-03-14

**Authors:** María José Calvo, Heliana Parra, Raquel Santeliz, Jordan Bautista, Eliana Luzardo, Nelson Villasmil, María Sofía Martínez, Maricamen Chacín, Clímaco Cano, Ana Checa-Ros, Luis D'Marco, Valmore Bermúdez, Juan Bautista De Sanctis

**Affiliations:** 1. Endocrine and Metabolic Diseases Research Center, School of Medicine, University of Zulia, Maracaibo, Venezuela; 2. Facultad de Ciencias de la Salud, Barranquilla, Universidad Simón Bolívar, Barranquilla, Colombia; 3. Research Group on Cardiorenal and Metabolic Diseases, Departamento de Medicina y Cirugía, Facultad de Ciencias de la Salud, Universidad Cardenal Herrera-CEU, CEU Universities, Valencia, Spain; 4. School of Life and Health Sciences, Aston University, Birmingham, United Kingdom; 5. Institute of Molecular and Translational Medicine, Faculty of Medicine and Dentistry, Palacky University, Olomouc, Czech Republic

**Keywords:** Foetus, gestational diabetes, insulin resistance, low-grade inflammation, obesity, placenta, quality of life

## Abstract

During pregnancy, women undergo several metabolic changes to guarantee an adequate supply of glucose to the foetus. These metabolic modifications develop what is known as physiological insulin resistance. When this process is altered, however, gestational diabetes mellitus (GDM) occurs. GDM is a multifactorial disease, and genetic and environmental factors play a crucial role in its aetiopathogenesis. GDM has been linked to both macroscopic and molecular alterations in placental tissues that affect placental physiology. This review summarizes the role of the placenta in the development of GDM from a molecular perspective, including hormonal and pro-inflammatory changes. Inflammation and hormonal imbalance, the characteristics dominating the GDM microenvironment, are responsible for placental changes in size and vascularity, leading to dysregulation in maternal and foetal circulations and to complications in the newborn. In conclusion, since the hormonal mechanisms operating in GDM have not been fully elucidated, more research should be done to improve the quality of life of patients with GDM and their future children.

Gestational diabetes mellitus (GDM) is generally defined as “any degree of glucose tolerance with onset or first recognition during pregnancy”.^1^ It currently is one of the diseases with the highest morbidity among pregnant women.^2^ Determining its prevalence has been a real challenge for the scientific community due to multiple modifications in the diagnostic criteria established in 1964 by O’Sullivan and Mahan.^3^ Globally, its prevalence ranges from 1% to 14%.^2^ Nevertheless, in a systematic review and meta-analysis providing updated estimates of gestational diabetes in Latin American in 2022, the reported prevalence was 8.5%.^4^

GDM is a multifactorial disease in which both genetic and environmental components play a crucial role in its aetiopathogenesis.^5^ It is characterized by the inability to compensate for the physiological insulin resistance (IR) generated by hormonal and inflammatory changes that normally occur during pregnancy.^6^ The hyperglycaemia that occurs in GDM has multiple consequences for both the mother and the foetus, not only during intrauterine life but also during childbirth, the perinatal period and beyond.^7,8^ About 50% of women diagnosed with GDM during pregnancy will develop type 2 diabetes mellitus (T2DM) in the future.^9^ Furthermore, foetuses from mothers with GDM can present with short-term complications, such as macrosomia, shoulder dystocia and neonatal hypoglycaemia. These children also have a greater risk of developing obesity and T2DM in adulthood.^6^

Many maternal and foetal complications of GDM have been attributed to abnormal placental development and anatomical and functional alterations.^10^ The placenta is a complex organ that separates the maternal and foetal circulations due to its anatomical configuration. As a result, it is exposed to multiple maternal and foetal substrates. Recent studies also highlight the role of certain regulators that are responsible for placental malfunctioning and GDM development.^11,12^ These studies mention several placental hormones, pro-inflammatory molecules, endothelial cell dysfunction and maternal adipose tissues, as well as the interruption of several molecular pathways, such as nuclear factor kappa-l ight-chain enhancer of activated B cells (NF-κB), peroxisome proliferator-activated receptors (PPARs), sirtuins, 5’ AMP-activated protein kinase (AMPK), glycogen synthase kinase 3 (GSK-3), inflammasome and endoplasmic reticulum stress.^13–15^

**Figure 1:**
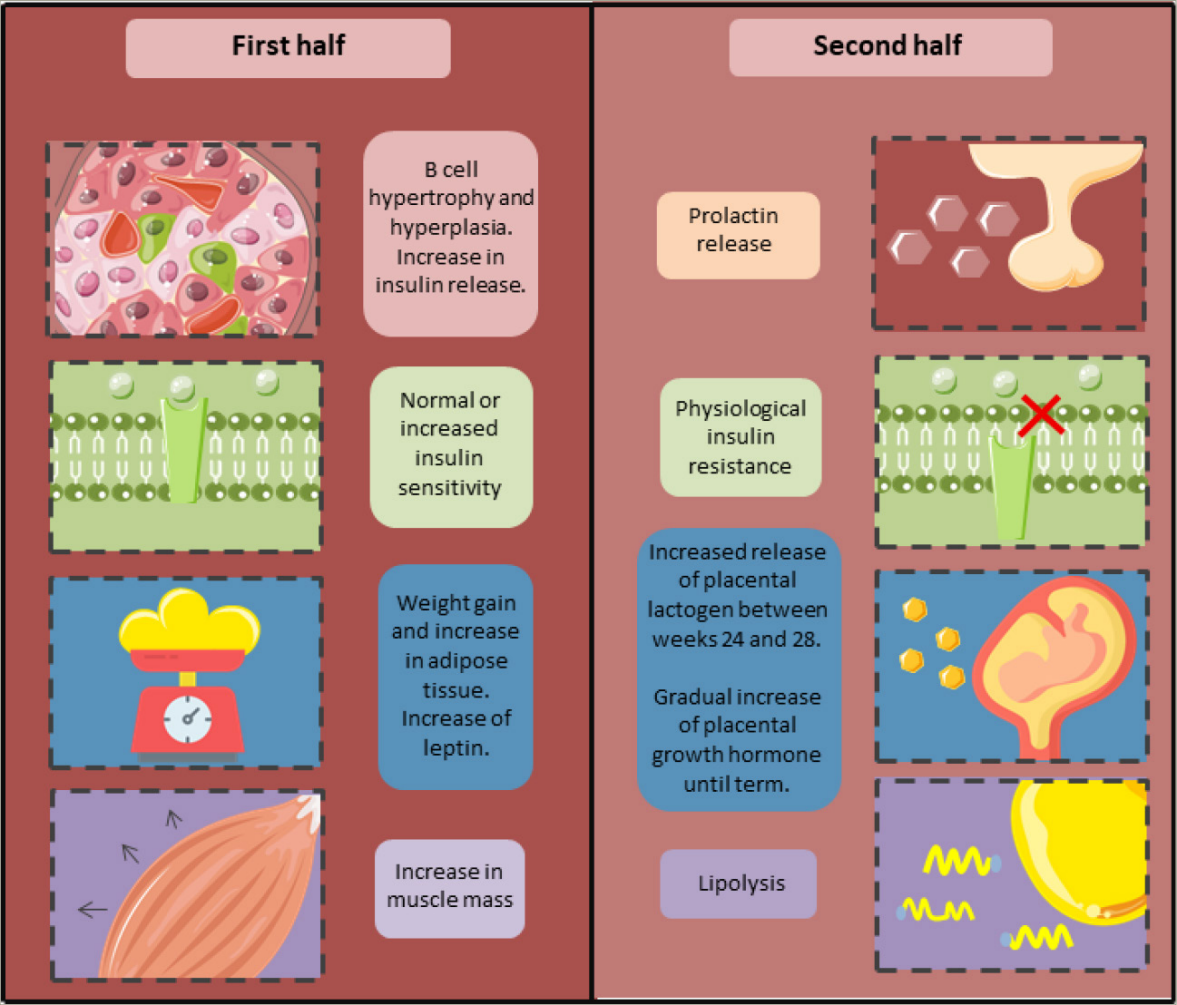
Physiological changes in macromolecule metabolism during pregnancy^16^

This review aims to illustrate the role of the placenta in the development of GDM from a molecular perspective. We start by describing the pro-inflammatory factors and changes in different hormones, such as the placental lactogen, PGH and adipokines, which precede the anatomical disturbances leading to alterations in the placental endothelial and angiogenic factors and transporters. Finally, we explore their impact on foetal development.

## From physiological insulin resistance to gestational diabetes: When equilibrium is lost

Glucose is the primary energy substrate needed for foetal and placental growth. Since foetal gluconeogenesis is minimal during gestation, pregnant women undergo several physiological changes in their metabolic, renal, immune, cardiovascular, haematologic and respiratory systems to guarantee a continuous and adequate supply of glucose to the foetus (Figure 1).^16,17^ Regarding metabolic changes, the onset of physiological IR, which increases as the pregnancy progresses, is necessary to originate a maternal–foetal glucose transfer system via facilitated diffusion, mainly through the glucose transporter 1 (GLUT1).^18,19^

In a normal pregnancy, there is an increase in insulin sensitivity during the first half of the pregnancy. Subsequently, a decrease in insulin sensitivity of up to 60% has been observed.^20^ This is followed by reversible compensatory changes in pancreatic beta cells, perhaps involving an increase in their number (hyperplasia) and size (hypertrophy) due to higher levels of oestrogen, progesterone, human placental lactogen (hPL), prolactin (PRL), kisspeptin and cortisol, among other growth factors.^21^

Both hPL and PRL exert their effects via prolactin receptors (PRLRs), which are specifically expressed in pancreatic beta cells. The intracellular mechanisms of these receptors involve the regulation of pro-proliferative and anti-apoptotic pathways via the Janus kinase 2-signal transducer and activator of transcription 5 (JAK2/STAT5) pathway, mitogen-activated protein kinase (MAPK), phosphatidylinositol 3-k inase (PI3K), and protein kinase B (AKT).^22^ Furthermore, there is an increase in glucokinase synthesis, resulting in a decrease in glucose-induced insulin secretion threshold, leading to hyperinsulinaemia, which allows the maintenance of euglycaemia during pregnancy.^19,23^ Studies show that the increase in hPL-dependent insulin secretion is mediated by increased levels of enzymes regulating serotonin synthesis, such as tryptophan hydroxylase 1 and 2.^24^ Likewise, there is hypertrophy and compensatory hyperplasia of pancreatic beta cells in the mother as a physiological adaptation to hPL pregnancy. When gestation ends, the pancreatic beta-cell mass starts to regress until it reaches normal conditions.^25^ Although the mechanisms responsible for these processes are not yet completely understood, it is known that, after childbirth, the beta-cell mass is reduced via apoptosis.^26^ Other studies suggest that this is due to a reduction in their size and proliferation.^27^ However, these processes are yet to be demonstrated in humans.^28^

In women who are obese, pancreatic beta cell dysfunction – including the inability of beta cells to undergo adaptive changes after the first trimester of pregnancy – may create a pro-inflammatory environment, which is capable of disrupting the appropriate response before the endocrine signs of pregnancy, increasing the risk of developing GDM.^29^ This phenomenon may be explained by an increase in platelet-derived growth factor because the levels of this peptide are inversely related to pancreatic beta-cell function in patients with GDM. On the other hand, it is believed that the chemokine (C-X-C motif) ligand 10 (CXCL10), an inflammatory marker, is capable of inhibiting the proliferation of pancreatic beta cells through binding to C-X-C motif ligand 3 (CXCL3), or through interaction with the toll-l ike receptor 4 as part of the NF-κB activation pathways mediating IR in GDM.^30^ Although an in-depth review of the immunological alterations in GDM is not within the scope of this work, these findings highlight the immune-related features mediating GDM pathogenesis.

In pregnant women, several factors increase the risk of a disturbance in physiological IR. These include family and personal history of diabetes mellitus, obesity, race (primarily indigenous people worldwide, African American and Hispanic),^23,24^ and advanced maternal age (≥35) (*Table 1*).^24,31^ As a result, IR becomes more severe and cannot be compensated by maternal hyperinsulinemia, resulting in the characteristic hyperglycaemia of GDM.^32^ The mechanism by which these alterations occur requires further study. However, it is believed that obesity causes a decrease in hPL levels via the downregulation of CCAAT-enhancer binding protein transcription factors, which are co-expressed with hPL in the syncytiotrophoblast (SCTB). C/EBP binds to an enhancer region downstream of the *hPL* gene, modifying its expression. Therefore, its downregulation results in deficiencies in compensatory metabolic effects.^33^

**Table 1:** Risk factors for gestational diabetes mellitus^31^

Low risk	High risk
Ethnic group with a low incidence of GDM	Being part of an ethnic group with a high incidence of GDM
No family history of DM	First-degree family history of DM
Younger than 25 years old	Previous detection of glucose intolerance
Normal weight	Obesity
Normal weight at birth	Glycosuria accentuated during pregnancy

*Adapted from Santamaria et al., 2011.^31^*

*DM = diabetes mellitus; GDM = gestational diabetes mellitus.*

**Figure 2:**
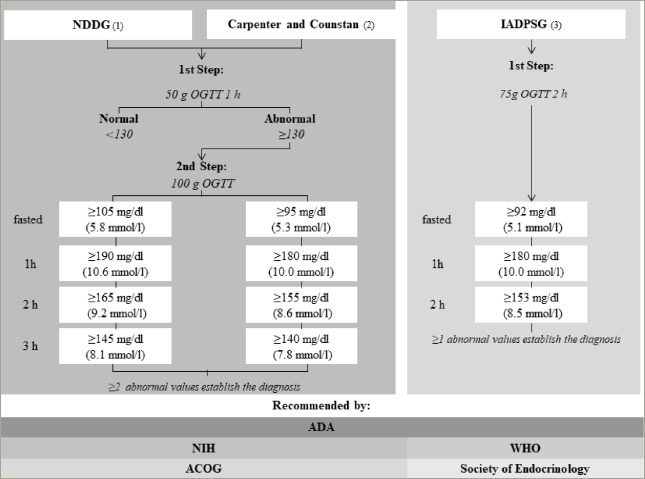
Different diagnostic criteria for gestational diabetes mellitus and recommending associations

*Algorithm formulated from diagnostic measures outlined in international guidelines from Harris, 1995;^34^ Carpenter and Coustan, 1982;^35^ International Association of Diabetes and Pregnancy Study Groups Consensus Panel, 2010.^36^*

*ACOG = American College of Obstetricians and Gynecologists; ADA = American Diabetes Association; h = hour; IADPSG = International Association of Diabetes and Pregnancy Study Groups; NGGD = National Diabetes Data Group; NIH = National Institutes of Health; OGTT = oral glucose tolerance test; WHO = World Health Organization.*

The incidence of GDM is higher among women with a higher body mass index (BMI) and weight before pregnancy compared with those with a normal BMI. A significant weight gain during the first trimester was positively associated with GDM in women with high BMI before pregnancy.^26^ These alterations are primarily seen in the last trimester. Hence, the diagnosis of GDM is made after week 24 using specific diagnostic criteria (*Figure 2*).^31,35,36^

## Placental hormones: When the good goes bad

### Placental lactogen and placental growth hormone

Since the mid 20th century, the main placental hormones, hPL and placental growth hormone (PGH), have been considered vital elements in the aetiopathogenesis of GDM, given their known diabetogenic effects in pregnant women.^36^ Nevertheless, even though the current evidence is not conclusive, it suggests a possible secondary role within the pathogenesis of this metabolic disease, with a greater emphasis on PGH dysregulation.^37^

PGH is a polypeptide drug synthesized in placental SCTB from gestational weeks 13 to 20, replacing the pituitary growth hormone from that moment onwards.^32^ Its release is independent of the growth hormone-releasing hormone and induced by maternal hypoglycaemia.^33^ The direct and indirect effects of PGH as a primary regulator of insulin growth factor 1 (IGF-1) are limited to maternal and placental tissues, regulating placental growth and blood flow and generating a state of physiological IR to reduce the maternal use of glucose so that it can reach the foetus and allow adequate growth and development.^31^ Nonetheless, insulin is not the only hormone able to activate these signalling pathways since cytokines, as well as other hormones and growth factors, play a very important role in their activation. These molecules are also involved in cellular metabolism and may affect the transport of nutrients.^38^

Among the mechanisms by which PGH generates a state of IR is a reduction in adiponectin synthesis. It is an important insulin-sensitizing and anti-inflammatory hormone, possibly via the activation of STAT5 proteins and their posterior binding to adiponectin promoter sites, decreasing its expression and release.^36^ Furthermore, it inhibits the insulin intracellular signalling pathway, with subsequent inhibition of glucose transporter 4 (GLUT4) translocation via the induction of expression of the gene-e ncoding *p85* subunits; this competitively inhibits insulin receptor substrate 1 (IRS-1), which activates PI3K by preventing its heterodimerization with a p110 subunit in insulin-dependent tissues.^39,40^ Thus, along with other diabetogenic hormones, this mechanism creates a diabetogenic environment that favours maternal catabolic processes that generate energetic substrates for the foetus. Although these processes occur physiologically, their role in GDM has been associated with an overproduction of PGH,^41^ demonstrating a possible dose–dependent relationship. However, the mechanisms causing these findings have not been completely elucidated.

Moreover, hPL is a polypeptide hormone synthesized by SCTB, whose levels increase significantly at the start of the second trimester. Its effects are mediated by PRLRs, activation of which mainly regulates the function and proliferation of pancreatic beta cells to maintain insulin release that compensates for the state of IR generated during gestation. Lepercq et al. proposed that polymorphisms of a single nucleotide (rs10068521 and rs9292578) in the gene encoding for PRLRs are related to a 2.3-fold increased risk of developing GDM.^42^

It was proposed that these effects are related to the downregulation of molecules that inhibit the cell cycle, such as the B-cell lymphoma 6 (BCL6) protein and cyclin-dependent kinase inhibitor 1 (p21), as well as the induction of anti-apoptotic molecules, such as B-cell lymphoma-extra-large (BCL-xL) and securin (PTTG1), and the protooncogene *FoxM1*.^43,44^ Thus, any alterations of these molecules in pancreatic beta cells or PRLRs may be involved in the reduction of proliferation and expansion of pancreatic beta-cell mass, leading to a deficient insulin release incapable of overcoming maternal IR and GDM development.^42,45,46^

### Leptin

Leptin has also been associated with the development of IR and GDM.^47^ This polypeptide hormone is mainly synthesized in white adipose tissues. However, during gestation, the SCTB constitutes the primary synthesizing tissue. Hence, leptin levels are higher than they are in non-pregnant women^48^ . These levels progressively increase after implantation, reaching a peak between weeks 24 and 26, and persist until the immediate postpartum period.^49,50^ During gestation, leptin regulates vital processes, such as implantation, mitogenesis and placental growth, as well as the placental transport of amino acids, the release of human chorionic gonadotropin, immune response and maternal appetite.^51,52^

Although a state of hyperleptinaemia is considered physiological, multiple studies reported significantly higher leptin levels in women with GDM.^53–55^ This could be the result of a combination of factors, including polymorphisms in the LEP rs2617270 allele of the gene that codes for this hormone, obesity prior to conception and excessive weight increase during pregnancy.^52,55–57^ These conditions are associated with IR and states of compensatory hyperinsulinaemia. Since insulin is a critical inductor of leptin secretion by white adipose tissues, it further stimulates its release, causing a vicious cycle. Moreover, despite the fact that leptin released by trophoblastic cells has a wide range of biological functions and plays a role in the successful establishment of pregnancy, it results in a higher hyperleptinaemia under these conditions, contributing to glucose resistance.^58^

In this sense, it was proposed that hyperleptinaemia during the first trimester could significantly increase the risk of developing GDM by further accentuating the state of physiological IR via the release of tumour necrosis factor-alpha and interleukin 6 (IL-6) in the placenta.^59^ These inhibit the insulin intracellular signalling pathway and decrease its peripheral effects.^60^ Furthermore, the hyperleptinaemia would increase lipid mobilization, accentuating the lipotoxicity states of peripheral tissues.^61^

### Resistin

Resistin is another adipokine that has generated interest over the last decades due to its association with IR, participating in entities such as T2DM, metabolic syndrome and obesity.^62,63^ This adipokine is a polypeptide hormone composed of 108 amino acids secreted by adipose cells during adipogenesis. Based on recent studies, resistin is also secreted by the pancreatic islets, skeletal muscle, mononuclear cells and liver, as well as the placenta, which is the primary source of resistin during gestation.^62,63^

Like the previously described hormones, resistin secretion is progressive, reaching its peak in the third trimester and returning to normality after childbirth.^64^ It was reported that women with GDM have higher serum resistin levels.^65^ These levels increase even further if there is obesity since, just as with leptin, supraphysiological insulin levels stimulate resistin release even further in the placenta and adipose tissues.^61^ On the other hand, case-control studies have not shown an association between resistin levels and GDM, suggesting that resistin does not predict risk for this disease.^44^

The implication of resistin in GDM development can be explained by its actions on the pancreatic islet, where it induces the expression of the suppressor of cytokine signalling 3 (*SOCS3*) and inhibits the phosphorylation of the AKT pathway, leading to a decrease in insulin release.^66^ Moreover, it reduces the insulin sensitivity of target tissues (skeletal muscle, adipose tissue and liver), affecting the translocation of GLUT4 and disturbing glucogenic metabolism.^67^ In the liver, resistin influences lipid metabolism by decreasing AMPK phosphorylation, which results in a decrease in beta-oxidation and increments in esterified fatty acids and triacylglycerides, leading to lipid accumulation in the liver parenchyma. However, given that the estimation of resistin has not been standardized, its relationship with GDM has not been completely elucidated.^68^

## Diabetes during the third trimester: From subtle to catastrophic placental changes

In GDM, there is a growing dysregulation of glucose metabolism, with the participation of a complex network of signalling molecules that result from a process of low-grade inflammation. When the third trimester is reached, this situation reaches its peak, and hyperglycaemia manifests. This results in micro-and macroscopic alterations in placental anatomy and function.^69^

### Anatomical alterations of the placenta

Macroscopically, at full term, the placenta is circular, with an approximate diameter of 22 cm, thickness of 2.5 cm and an average weight of 470 g.^70^ These characteristics vary from one placenta to another. The placenta has two surfaces: the foetal (chorionic plate) and the maternal side (basal plate or decidua). The latter comprises a mixture of basal decidua and trophoblastic cells and contains placental cotyledons or lobes. Between the chorionic and decidual plate, there is an intervillous space divided by incomplete septa formed by extravillous trophoblast, decidual cells and fibrinoid tissue. Furthermore, there are villi extending from the chorionic plate. These are covered by the placental membrane or barrier, constituted by the cytotrophoblast (CTB) and the SCTB; these can be classified into floating, anchoring or exchange villi, depending on their function.^70^ Floating villi are subdivided according to their stromal characteristics and calibre into mesenchymal villi (rich in mesenchymal cells), immature intermediate villi (with stromal channels containing foetal macrophages or Hofbauer cells), stem villi (the largest ones, with a perivascular contractible system around its main vessels), mature intermediate villi (only contain vessels and capillaries within loose stroma), and terminal villi (possess sinusoids and capillaries with a thin vasculosyncytial membrane).^71^

In contrast, women with uncontrolled GDM have bigger placentas. This organ becomes thick and plethoric, which can explain the significant increase in placental weight. Studies reported that the placentas of patients with GDM are heavier compared with those from normoglycaemic controls.^72^ The placentas of pregnant women diagnosed with GDM are 22% heavier and have 33–85% bigger diameter and central thickness, respectively, compared with normal placentas.^72^

Microscopically, fibrinoid necrosis, vascular lesions, an increase in blood vessels within chorionic villi, oedematous intima, fibrin deposits in SCTB, focal calcifications, Hofbauer cell hyperplasia, syncytial nodes and marked CTB hyperplasia are also attested. It is relevant that the vasculosyncytial membrane of terminal villi has an oedematous stroma, increased syncytial nodes and fibrin deposits. These changes can negatively affect vascular transport, leading to hypoperfusion; this increases the risk of placental infarction, villous immaturity and decidual vasculopathy.^72,73^

**Figure 3:**
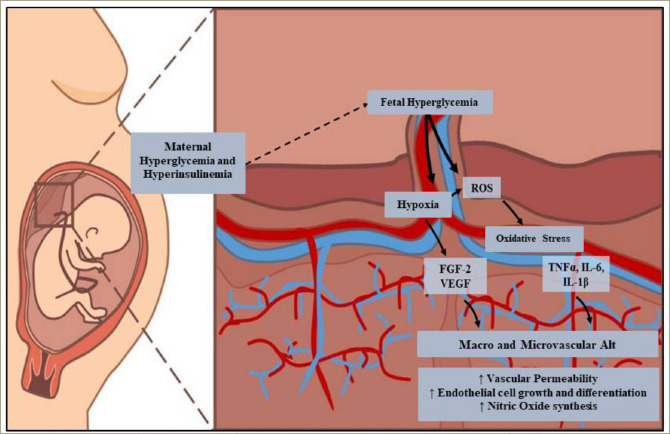
The macrovascular and microvascular alterations of the placenta occur due to maternal hyperglycaemia and compensatory hyperinsulinaemia, which lead to foetal hyperglycaemia

*Alt = alterations; FGF-2 = fibroblast growth factor-2; IL = interleukin; ROS = reactive oxygen species; TNFα = tumour necrosis factor-alpha; VEGF = vascular endothelial growth factor.*

Trophoblastic proliferation has been described as uncontrolled, with cellular incoordination due to alterations in placental growth factors, such as vascular endothelial growth factor (VEGF) and colony-stimulating factor.^74^ This results from a hypoxic environment emanating from hyperglycaemia due to the activation of pathways, such as the PI3K/AKT1 pathway.^75^ An increase in capillary surface and blood vessel penetration was also observed in the placentas of women with GDM due to higher oxygen demand, with significantly more villous CTB, fibroblasts and macrophages.^76^ Moreover, a reduction in the apoptotic index was observed in the placentas of pregnant women with GDM, which was associated with an increase in maternal weight.^77^ Finally, regarding the trophoblastic cell cycle, the metabolic environment of GDM affects the expression of genes that control the cell cycle, such as *p27* and *p57*. The latter is a tumour suppressor and an inhibitor of the cell cycle. Its loss leads to a lack of cell cycle control and hyperproliferation. Hence, a lower expression of this gene results in large and heavy placentas in GDM.^76^

### Placental vascular alterations

The vascular integrity of the placenta allows normal foetal development. This situation is altered in women with GDM due to a hyperglycaemic environment, which generates alterations in the macrovasculature (i.e. human umbilical venous and arterial endothelial cell), and the microvasculature of the human placenta (i.e. human placental microvascular endothelial cells) (Figure 3). The hypervascularization resulting from an increase in angiogenesis plays a fundamental role in the development of this disease.^78^ Studies reported that hyperglycaemia, hyperinsulinaemia, dyslipidaemia and hypoxic uterine environment are the causes of VEGF and fibroblast growth factor 2 overexpression.^75,79–81^ When VEGF binds to its receptor, it induces the self-phosphorylation of cadherin, which causes interruptions in the adherens junctions of endothelial cells, leading to increased vascular permeability.^81,82^ Although data on angiogenic factors in GDM are limited, there are recent studies suggesting that VEGF overexpression would be secondary to an increase in the angiogenic placental growth factor, which increases cell sensitivity to VEGF.^83–85^

Furthermore, L-arginine transport and nitric oxide (NO) synthesis increase due to hyperinsulinaemia.^86^ Insulin binds to the insulin receptor isoform A, triggering the p42/44 MAPK signalling pathway, which induces cell growth and differentiation, increasing hENT2 transporters (L-arginine transport). Insulin also activates the insulin receptor isoform B, which increases D-glucose metabolism and NO synthesis. In the absence of the disease, both signalling pathways maintain equilibrium in endothelial cells, but NO is also a free radical that has been associated with endothelial dysfunction.^86^ It is suggested that the GDM-related increase in NO production may have a potential role in the early stages of endothelial dysfunction in these patients.^87^

Moreover, in normoglycaemic patients, there is a balance between placental mucin 1 (*MUC1*) and adrenomedullin (ADM) in the promotion of placental growth and invasion. ADM is a negative regulator of *MUC1* gene expression in trophoblastic cells.^88^ However, due to the hypoxia caused by vascular alterations in the placenta, *MUC1* overexpression has been described.^89^ The role of ADM in early gestation has also been studied, suggesting that it regulates the optimal expression of *MUC1* to promote implantation and facilitate TB invasion.^90^

### Alterations in placental transport

Foetal development is closely related to the ability to transport nutrients to the foetus via maternal–foetal circulations.^59^ Illnesses such as GDM and obesity cause a positive energy balance, increasing the insulin concentration in the blood and triggering a dysregulation of growth factors, such as IGF-1, increasing the risk of foetal overgrowth.^91^

According to a study by Jansson and Powell, the placenta acts as a nutrient sensor.^92^ When the availability of nutrients decreases or is limited, foetal growth slows down or is restricted. On the other hand, there is an increase or acceleration of growth when there is an excess of nutrients. There is also an alternative hypothesis, which suggests that the placenta may respond in a compensatory fashion to the regulations in the activity of transporters upon high or low nutrient levels to maintain normal foetal growth.^93^

It is known that there is an alteration in placental function in response to maternal obesity. Hence, studies have shown that the capacity of the placenta regarding the transport of amino acids and lipids is greater in women with obesity; likewise, passive permeability of the placenta and glucose transporters are observed to be upregulated in parallel with increased foetal growth.^94,95^ These placental changes are discussed further below.

Lipids are found in the maternal circulation as fatty acids and triacylglycerides, which cannot penetrate the SCTB. Therefore, they must be hydrolyzed by lipoprotein lipase (LPL). LPL and endothelial lipase (EL) are expressed in microvilli, and their activity is influenced by cytokines, insulin, IGF-1, and leptin.^95^ One *in vitro* study showed that high IL-6 levels can cause fatty acid accumulation in trophoblasts ^96^ . Hence, cytokines can regulate lipid metabolism in the placenta.^97^ However, the elevation of these cytokines does not modify the function of fatty acid transporter proteins, adipophilin or the activity of LPL.^97^

Furthermore, studies with placental endothelial cells have shown an increased EL expression in the placentas of patients with obesity and GDM, which suggests that low-grade inflammation and other diabetogenic conditions generate a dysregulation of EL during gestation, where leptin and tumour necrosis factor-alpha have a key regulating role.^36,98^ Both cytokines contribute to an increase in the placental regulation of phospholipase A2 family members, leading to the accumulation of omega-3 polyunsaturated fatty acids in placental tissues.^45,49^ In this sense, omega-3 polyunsaturated fatty acids are widely known for their benefits in maternal and foetal regulation of metabolism, inflammation, oxidative stress and disorders such as foetal macrosomia and preeclampsia; in patients with GDM, these processes are highly compromised, which interrupts normal transport, resulting in reduced foetal levels, contributing negatively to all the aforementioned factors.^99^

Moreover, the transport of amino acids is also altered in GDM. It was proven that there is an increase in the transport and concentrations of essential and non-essential amino acids.^100^ The most studied amino acid transport systems are system A and system L.^100^ Alanine, glycine and serine are transported by amino acid transport system A, which is highly stimulated by insulin, leptin, IGF-1, and IL-6 and are present in the SCTB (especially in microvilli). On the other hand, amino acids with high molecular weights, such as leucine, are transported by amino acid transport system L, which is highly stimulated by glucose and insulin, and is found in microvilli and on the basal membrane.^95^

Lastly, glucose is one of the primary energy substrates for foetal and placental growth. Since glucose production in the foetus is minimal, the foetus is dependent on maternal glucose. According to a study on placental perfusion, there are no differences in the flow of glucose that enters the foetus through a concentration gradient from the placenta, independently of a GDM diagnosis.^101^ Furthermore, evidence supports the notion that the placenta is not related to the transference of maternal glucose to the foetus in GDM, and the concentration gradient is the critical point in this pathology.^101,102^ Nevertheless, an increase in placental size increases glucose transport, contributing to the accumulation of foetal fat in GDM.^59^ GDM and obesity are associated with high levels of pro-inflammatory cytokines, which could alter the expression of genes involved in lipid pathways and contribute to the increase in placental lipid transport.^103^

## The effect of placental changes on foetal anthropometric measurements

The alterations in an intrauterine environment that arise from the previously described metabolic and inflammatory disorders generate a series of foetal complications. The most important are foetal macrosomia and decreased muscle mass.

### Foetal macrosomia

Foetal macrosomia is one of the most common complications of GDM, affecting approximately 15–45% of infants born to patients with this disease.^104^ Foetal growth is a complex process involving genetic, endocrine and metabolic factors. Therefore, any alterations in one of these factors in the mother, placenta, or foetus can alter foetal growth or result in foetal macrosomia. This condition is defined as a birth weight of 4,000 g or higher. It can be symmetrical or asymmetrical, with the latter being evidence of a diabetic pregnancy. It is characterized by an increase in the abdominal diameter and scapular area of the foetus, in contrast with the dimensions of the head and femur.^105^

**Figure 4:**
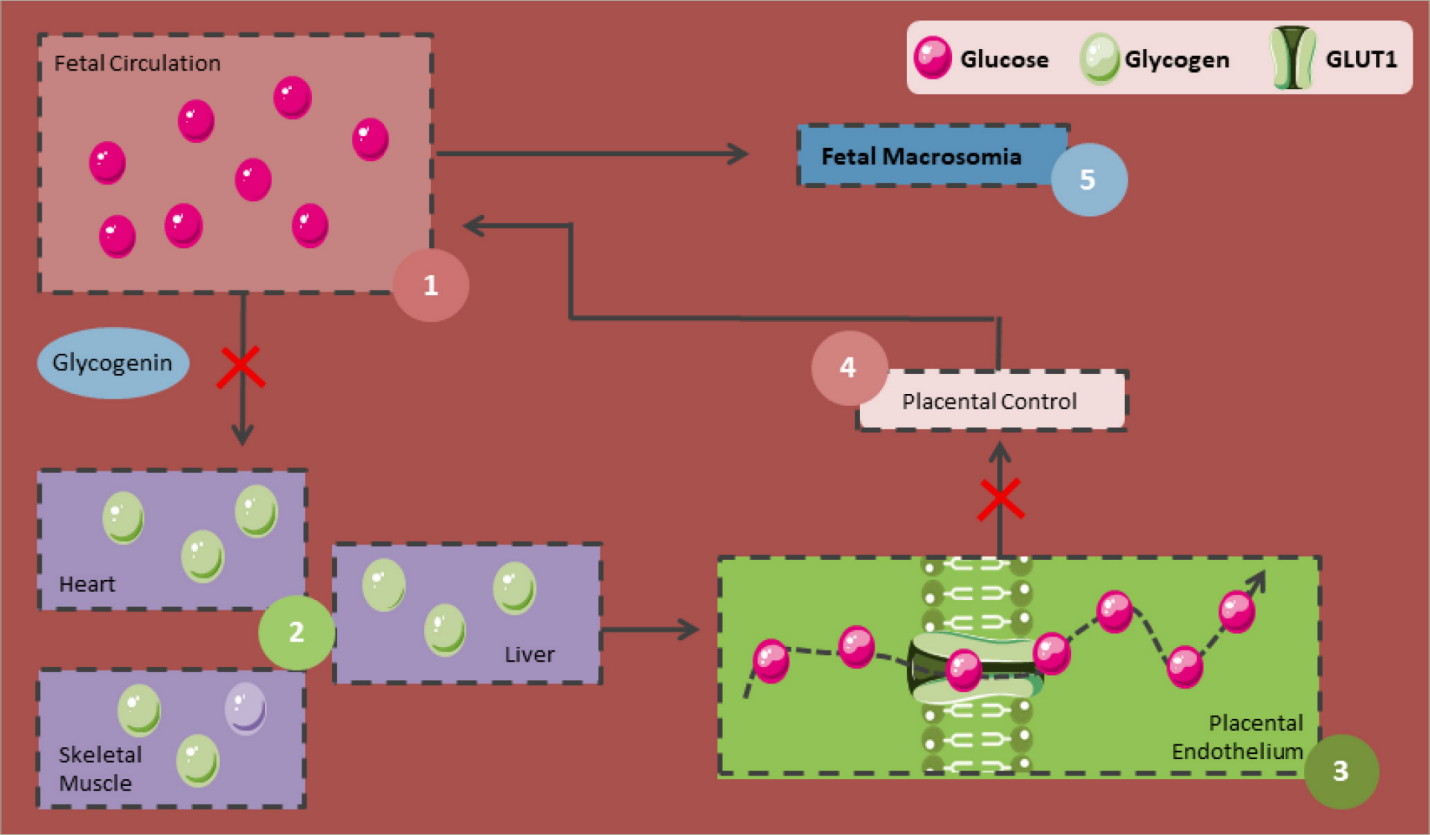
Physiopathology of foetal macrosomia

Although its underlying mechanisms are not entirely understood, foetal macrosomia can be explained by the Pedersen hypothesis, where maternal hyperglycaemia leads to foetal hyperinsulinaemia and an increase in the adipose tissue (Figure 4). When maternal hyperglycaemia is present, glucose crosses the placenta in higher amounts and is stored as glycogen. However, once these glycogen deposits become saturated, the excess glucose and the corresponding foetal hyperinsulinaemia, along with the increase in growth factors similar to insulin, lead to an increase in fat and protein deposits, resulting in macrosomia.^106,107^

Foetal macrosomia is also induced by a placental increase in certain genes in mothers without GDM, such as placental expression of *PPAR alpha and gamma*, which are considered transcriptional regulators with key functions in lipid metabolism and *miR-27b*.^108^ On the other hand, the effects of long non-coding RNAs (lncRNA) in non-diabetic foetal macrosomia have also been studied, suggesting that the lncRNA *USP2-AS1* promotes placental development by affecting the proliferative activity of placental cells.^109^ Moreover, another study indicated that lncRNA-SNX17 overexpression reduces the expression of *miR-517a*, which could play an important role in the regulation of birth weight, and increases the expression of IGF-1 in the human trophoblast cell line HTR-8/SVneo, thus enhancing the proliferation of HTR-8/SVneo.^110^ It also reported that *lncRNA-SNX17* could promote trophoblast proliferation through the miR-517a/IGF-1 pathway and could play a role in diabetic macrosomia placentation.^110^ However, further studies are required to elucidate the underlying mechanisms.

Infants born to women with GDM are susceptible to abnormal foetal growth, even those with normal glucose levels.^111^ This may be explained by the role of foetal IGF-1, which regulates foetal growth and is increased by about 55% during gestation in women with GDM. It is believed that IGF-1 stimulates macrosomia via mammalian target of rapamycin (mTOR) activation and the subsequent increase in essential and non-essential amino acid transporters in the placenta of women with GDM.^112^

The overgrowth pattern typically seen in macrosomic newborns is known to be one of asymmetrical growth, with central deposition of subcutaneous fat around the abdomen and back, greater shoulder circumference and increased skin folds.^113,114^ However, no studies have been conducted to confirm the shape of fat mass distribution in macrosomic newborns.^115^ Anthropometric indices have also been established for the early diagnosis of foetal macrosomia, among which the maternal Body Roundness Index, Body Shape Index and Visceral Adiposity Index stand out. In a study conducted by Ozler et al., the efficacy of these anthropometric indices in obese and non-obese pregnant women was investigated; the results found that these were significantly higher in the group of women who were obese who subsequently had a macrosomic newborn.^116^

*GLUT1 = glucose transporter 1.*

**Figure 5:**
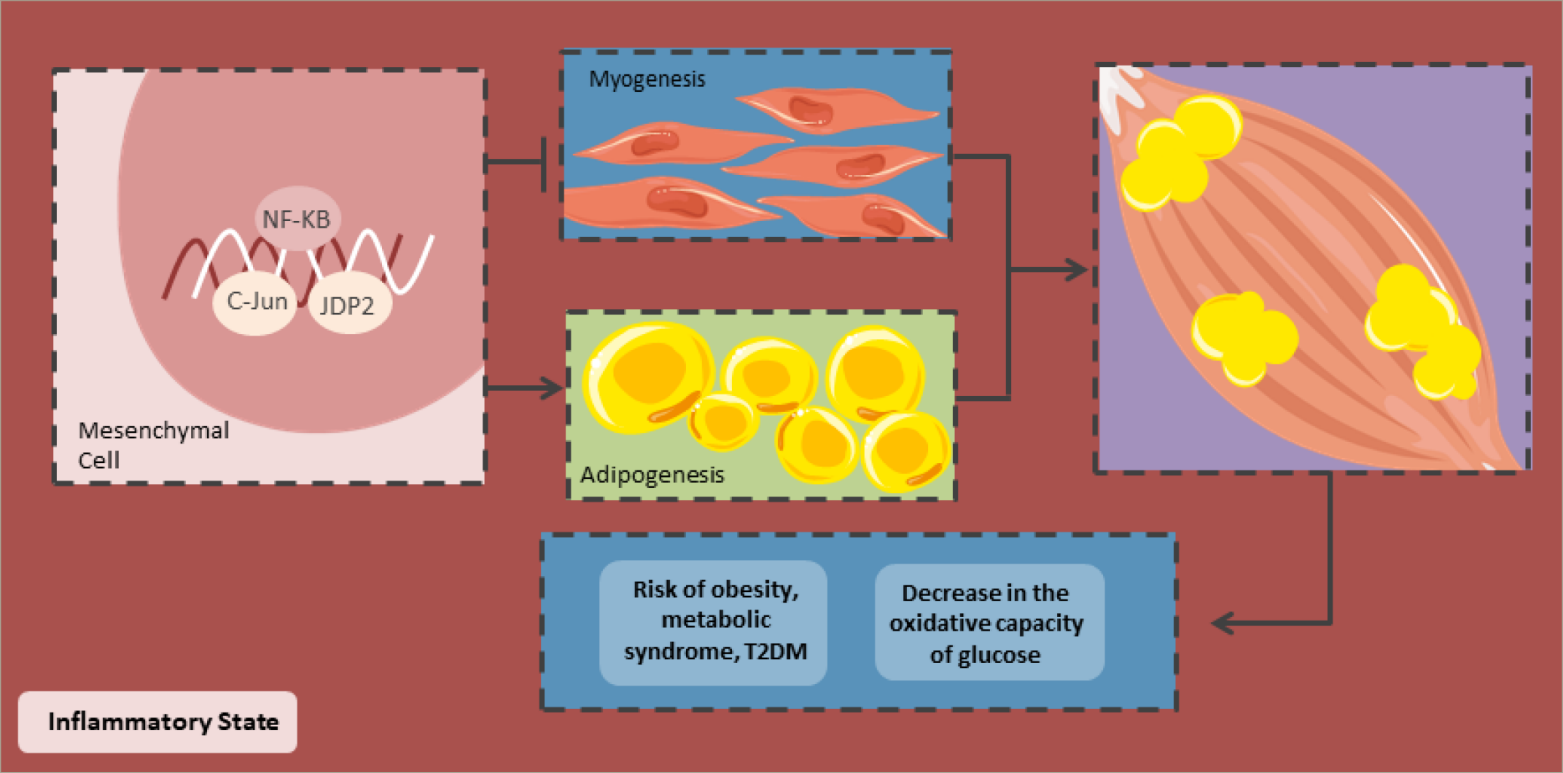
Decrease in the oxidative capacity of skeletal muscle

*C-Jun = transcription factor Jun; JDP2 = Jun dimerization protein 2; NF-κB = nuclear factor kappa-light-chain enhancer of activated B cells; T2DM = type 2 diabetes mellitus.*

### Muscle mass

Due to an altered cytokine release, low-grade inflammation can lead to changes in foetal muscle development (Figure 5). Inflammation promotes adipogenesis and activates NF-κB to differentiate adipocytes and decrease myogenesis.^117^ Furthermore, the differentiation of mesenchymal cells in muscle tissues is diminished due to the dimerization of *c-Jun* with the JDP2 protein, which inhibits the transcriptional activity of Jun, inhibiting myogenesis. Thus, a low-grade inflammation decreases myogenesis and increases adipogenesis in foetal skeletal muscle, decreasing its oxidative capacity. Hence, the newborn is at greater risk of having IR, obesity and T2DM in the long term.^118^

**Table 2:** Hormonal disturbances and alterations in placental transport and growth occurring in gestational diabetes mellitus and the impact on the foetus

Feature	Women without GDM	Proposed pathophysiological effects on the foetus
Hormones	Elevated insulin (insulin resistance) Impaired glucose tolerance Elevated adipokines	Foetal overgrowth (increased risk of macrosomia) Risk of foetal hypoglycaemia due to increased insulin secretion
Placental growth	Abnormalities in placental growth and vascular development Reduced placental perfusion	Foetal hypoxia Foetal growth restriction contributing to IUGR
Placental transport	Altered nutrient and oxygen transfer	Foetal developmental delay IUGR Increased risk of metabolic abnormalities

*GDM = gestational diabetes mellitus; IUGR = intrauterine growth restriction.*

It has been postulated that the overexpression of PGH increases the expression of *p85* subunit proteins of PI3K in insulin-sensitive tissues (i.e. the skeletal muscle).^119^ This decreases its signalling and leads to IR. Furthermore, the IR induced by PGH results in reduced GLUT4 expression and decreased glucose uptake, which would lead to muscle mass loss.^120^

The hormonal disturbances and alterations in the placental transport and growth occurring in GDM and described above are summarized in *Table 2*.

## Conclusion

GDM is characterized by the inability to compensate for the physiological IR of pregnancy. Many factors have been associated with the development of IR during gestation. Placental hormones, along with the increase in the production of pro-inflammatory cytokines, maintain the characteristic low-grade inflammatory state of GDM, which is the cause of morphological and functional changes in the placenta. The placenta plays a crucial role in GDM by functioning as a regulator of maternal and foetal circulations. Hence, changes in its size, vascular lesions and microscopic necrosis would affect this regulation. These changes result in several foetal complications, including foetal macrosomia. This condition originates from alterations in placental transport and a decrease in muscle mass, which results from a constant inflammatory process that decreases myogenesis and increases certain lactogenic hormones. Since the mechanisms by which these hormones interact in this pathology have not been fully elucidated, more research should be done to improve the quality of life of patients with GDM and their future children.
